# Cross-cultural translation and linguistic validation of the eating motivation survey among older adults in the Chinese context

**DOI:** 10.3389/fnut.2025.1610598

**Published:** 2025-07-30

**Authors:** Lina Wu, Xi Chen, Hui Feng, Shouzhen Cheng

**Affiliations:** ^1^The First Affiliated Hospital of Sun Yat-sen University, Guangzhou, China; ^2^School of Nursing, Sun Yat-sen University, Guangzhou, China; ^3^Xiangya School of Nursing, Central South University, Changsha, China

**Keywords:** eating motivation, food choice, linguistic validation, cognitive interview, healthy aging

## Abstract

**Background:**

Diet quality is a determinant of healthy aging and contributes to reducing the risk of chronic diseases such as cardiovascular disease, diabetes, and cancers, which impose considerable burdens on healthcare systems in China. Despite significant nutritional guidelines, older adults’ adherence to healthy eating remains inadequate. Understanding the determinants is essential for improving their adherence. Eating motivation is a key factor in exploring the reasons behind food choices.

**Methods:**

Brislin’s classical translation model was rigorously utilized to translate the Eating Motivation Survey (TEMS) into Chinese, involving forward translation, back translation, and expert consultation by a panel of healthcare and nutrition specialists. Cognitive interviews were conducted for further adaptation to assess clarity, intelligibility, and cultural appropriateness, following the Cognitive Interviewing Reporting Framework (CIRF). After linguistic adaptation, the Chinese version of TEMS was used on eligible older adults to test the reliability and validity.

**Results:**

Cognitive interviews conducted with 23 participants over 3 iterative rounds revealed issues with item wording, font size, and layout. Additionally, 249 elderly community residents participated in testing the reliability and validity. A total of 12 items were reworded to adapt the instrument to Chinese culture while maintaining their conceptual objectives. Colloquial words were revised and the formatting was adjusted with 1.5 line spacing and a font size of 14 in SimSun font to enhance readability. Practical examples were added to improve item comprehension, particularly for less-educated respondents. The results indicated that Cronbach’s *α* coefficient was 0.772, and the split-half coefficient was 0.871.

**Conclusion:**

This study successfully adapts TEMS to the Chinese context, providing a reliable and culturally sensitive measure of eating motivations, which is crucial for developing effective dietary interventions to enhance diet quality and promote healthy aging. The study underscores the importance of considering linguistic and cultural nuances in cross-cultural instrument adaptation and offers insights for future studies.

## 1 Introduction

**Figure 1:**
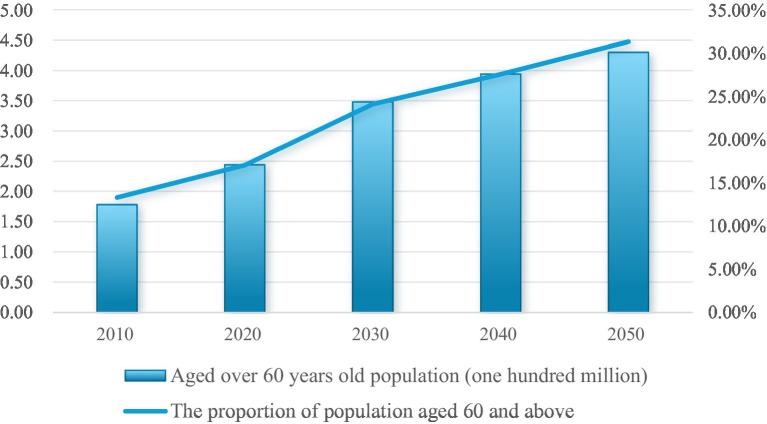
Trend of aging population in China.

The global demographic shift toward an increasingly aging population is characterized by a growing proportion of older adults and a corresponding increase in age-related diseases. This demographic transition underscores the importance of “healthy aging,” a paradigm aimed at minimizing the risk of age-related diseases and mitigating disability in advanced age (1), particularly in China (Figure 1), which has the largest elderly population in the world (2). Diet quality serves as a fundamental determinant of healthy aging (3), as unhealthy diets significantly contribute to obesity and chronic diseases (4, 5). Obesity, in turn, exacerbates these conditions and accelerates the transition to a poorer nutritional status (6).

Healthy eating can potentially prevent, treat, and even reverse chronic diseases (7–9), and it can contribute to the prevention of age-related syndromes such as frailty and sarcopenia, which significantly impair healthy aging (10, 11). Despite considerable research and nutritional guidelines, adherence to healthy eating among Chinese older adults remains inadequate (12, 13). The prevalence and mortality rates of chronic diseases are on the rise in China (14–16), which impose substantial burdens on healthcare systems (17). Effective measures are urgently needed to intervene in Chinese older adults’ unhealthy eating behaviors and improve their health condition. Identifying the underlying determinants influencing eating behaviors is essential for developing effective interventions to enhance dietary adherence and overall health, promoting healthy aging. One approach concerning individual drivers of eating behavior is to investigate their eating motivation (6). Eating motivation plays a pivotal role in shaping food choices, causes changes in the dietary patterns of older adults, and affects their nutritional health status (18, 19), which is considered a critical approach to understanding “why we eat what we eat” and comprehensively analyzing the determinants of eating behaviors (20, 21). Therefore, identifying the eating motivation characteristics of the elderly may help in the in-depth analysis of their eating behaviors and facilitate targeted measures in the future.

Multidimensional motivations influence older people’s daily lives and the cultural background that underpins their eating behaviors. Previous researchers have designed questionnaires to systematically assess individuals’ eating motivations. A pioneering effort in this field was the development of the Food Choice Questionnaire (FCQ) by British scholars Steptoe, Pollard, and Wardle in 1995 (22). However, the FCQ’s origins date back to a time when certain critical motivations, such as social and psychological factors, were not completely recognized (6). Subsequent research suggests that older adults eating behaviors are influenced by a complex interplay of personal emotions (23), social relationships, and other factors (24). To address these gaps, the Eating Motivation Survey (TEMS) was developed by a German research team in 2012 after a thorough review of previous quantitative instruments for measuring eating motivations, and 12 experts in nutrition and psychology were invited to be interviewed repeatedly, and 94 motivations were extracted from 241 kinds of motivations for compilation and factor analysis (21). Eventually, TEMS revealed 15 basic motivations for eating behaviors in daily life: likes, habits, needs and hunger, health, convenience, pleasure, traditional eating, natural attention, sociability, price, visual appeal, weight control, emotional regulation, social norms, and social image. TEMS has been recognized as a comprehensive and multifaceted tool (11) and has been widely applied in the United States (25), the United Kingdom (26), and India (19), among others, demonstrating satisfactory reliability and validity.

To our knowledge, there are currently no suitable instruments available to effectively evaluate the eating motivation of older Chinese adults. Our study aims to address this issue by translating and culturally adapting the TEMS to assess the eating motivations of this demographic. This effort is crucial for developing targeted interventions in future research that could improve adherence to healthy eating, enhance nutritional health, and reduce the burden of chronic diseases (27, 28). However, given that previous studies have been conducted in English, the interpretation and comprehension of TEMS items within the Chinese context remain uncertain. Chinese traditional culture possesses distinct characteristics when compared to those of other nations, such as its unique traditional values (“Mianzi” consciousness) (29). Given the linguistic and cultural subtleties, cognitive interviewing plays a crucial role in tailoring the TEMS to the Chinese context, in line with the recommendations of the Consensus-based Standards for the Selection of Health Measurement Instruments (COSMIN), to enhance the reliability and validity of questionnaires during cross-cultural adaptation (30, 31).

Hence, the primary goal of our study is to refine the TEMS for the Chinese context by identifying problematic items, assessing cultural appropriateness, and ensuring user-friendliness to validate its linguistic adaptation. Our study also seeks to confirm the cross-cultural applicability of TEMS.

## 2 Materials and methods

The TEMS original items were translated into Chinese by a panel of healthcare specialists and nutrition specialists, following Brislin’s classical translation model, a comprehensive recommendation of translation, adaptation, and validation of a model for cross-cultural research (32). This study utilized a sequential exploratory mixed-methods design. After the translation finished, cognitive interviews were conducted to ensure linguistic validation, following the Cognitive Interviewing Reporting Framework (CIRF) during the implementation and results report (33). Cognitive interviewing is a recommended method for pre-testing self-report tools to improve their reliability and validity by COSMIN, especially in cross-cultural adjustment and revision of the scale (30, 31). Subsequently, a cross-sectional survey was conducted to validate the Chinese version of the scale.

### 2.1 Study design

#### 2.1.1 Step 1: forward translation

**Figure 2:**
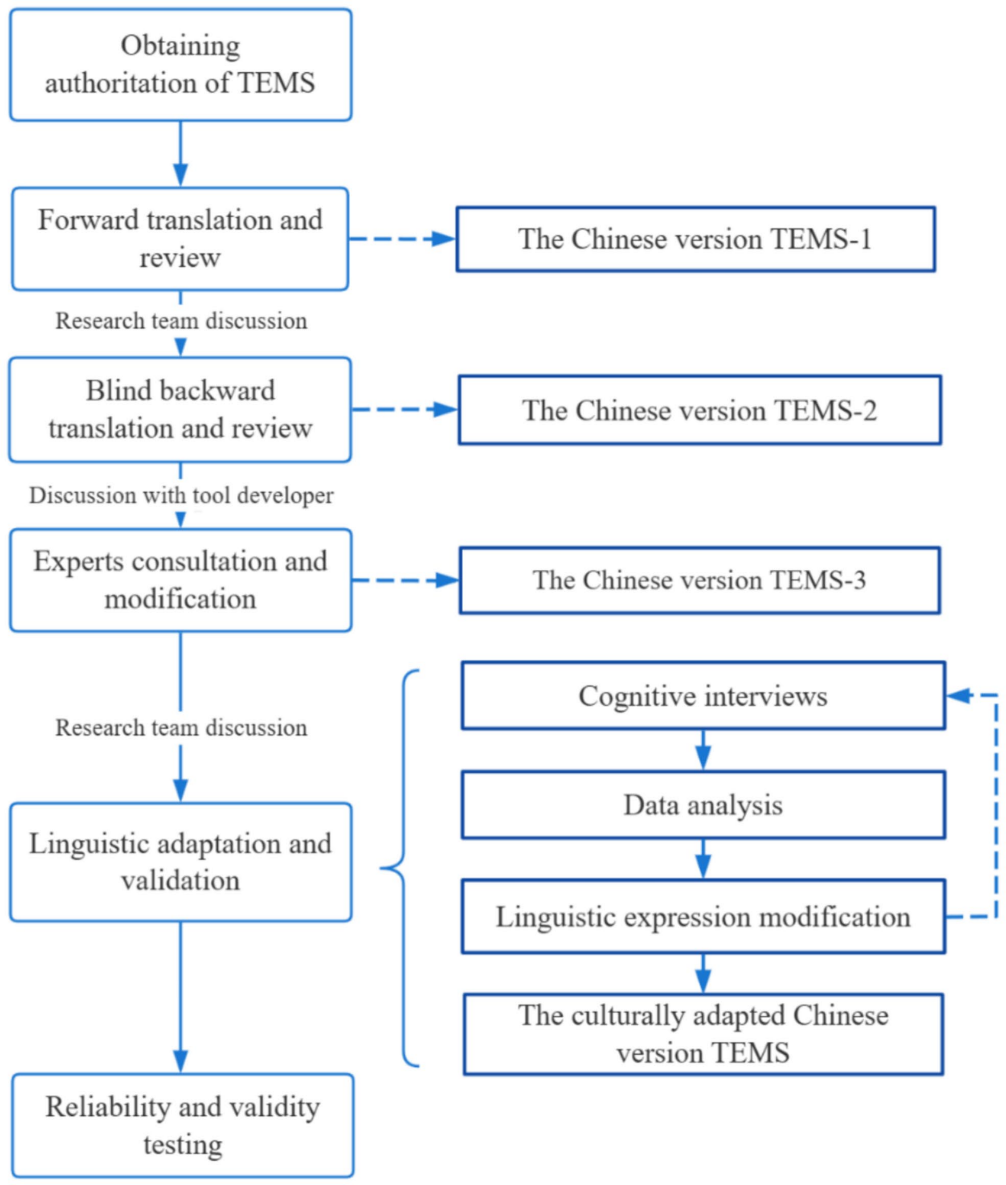
Illustration of the proceedings in the translation and linguistic validation of TEMS.

Two bilingual translators independently translated the original TEMS items and instructions into Chinese (Figure 2). One translator was knowledgeable about healthcare terminology and was fluent in English reading and speaking with 15 years of English study experience (WL). The other translator was a certified professional translator who graduated from Xiamen University and is an American native English speaker who holds a master’s degree in English translation (MW).

#### 2.1.2 Step 2: review the two forward translation versions

The research team members reviewed the two forward translation versions with the original English TEMS, evaluating whether the translation correctly reflected the intended meanings of the original. Any questions that the researchers could not resolve were discussed with the research team until a consensus was reached, and the two forward translation versions were eventually integrated and then modified into the preliminary Chinese version (TEMS-1).

#### 2.1.3 Step 3: blind backward translation

The TEMS-1 Chinese version (post-forward translation) was then independently translated back into English by two translators. One translator was a doctoral student at Xiangya Nursing School of Central South University and was familiar with healthcare terminology (JD). The other was a doctoral student at Shanghai Fudan University and has strong proficiency in English language (TOEFL score of 117) with 3 years of overseas study experience (TR). The backward translators had no previous exposure to the original English TEMS.

#### 2.1.4 Step 4: review the two backward translation versions

Similar to the forward translation comparisons, the research team reviewed the two back-translated versions with the original scale and re-translated the items with a concordance rate of less than 90% until the concordance rate reached more than 90%, forming the English back-translated version. A remote video was then conducted with the tool developer to review and determine if it had semantic and conceptual ambiguities (GS). The research team continued to revise according to the advice of the tool developer until a consensus was reached, forming the preliminary Chinese translated version (TEMS-2).

#### 2.1.5 Step 5: expert consultation’

Eight experts in geriatric nursing, geriatrics, and nutrition were invited to evaluate and modify the expression and semantics of the preliminary Chinese version TEMS-2 (Table 1). According to the expert recommendations, the revised Chinese version TEMS-3 was obtained after modification.

**Table 1:** Experts’ basic information.

Number of experts	Gender	Educational degree	Major	Professional title	Working years
A	Female	Master	Geriatric nursing	Senior nurse	22
B	Female	Master	Clinical nursing	Senior nurse	23
C	Female	Master	Clinical nursing	Co-chief nurse	22
D	Female	PhD	Geriatric nursing	Assistant professor	5
E	Female	PhD	Geriatric nursing	Professor	35
H	Female	PhD	Social medicine	Associate professor	8
F	Male	PhD	Nutrition	Professor	30
G	Male	PhD	Nutrition	Associate professor	22

The inquiry contents included the following: (1) whether the Chinese translation had the same meaning as the original, (2) whether the wording was used appropriately, (3) the relevance of each item to the assessed content, and (4) whether the expression habits conformed to Chinese culture.

Experts’ selection criteria included the following: (1) having an intermediate professional title and above or master’s degree and above, (2) having rich clinical experience in geriatric medicine or nutrition for 5 years and above, and (3) having abundant knowledge of the development of tools for measuring psychometric characteristics.

#### 2.1.6 Step 6: cognitive interviews

Linguistic validation assesses how participants understand and respond to the instruments, evaluating the target population’s clarity, intelligibility, appropriateness, and cultural relevance to the target language version (34). Cognitive interview is an effective approach to understanding cognitive biases between researchers and respondents (30). Verbal probing was utilized to conduct linguistic validation, a cognitive interview method. Simultaneously, the research team developed the cognitive interview outline through a comprehensive literature review, categorizing it into five probe categories (Table 2). For cognitive interviews, multiple rounds were conducted, with at least five interviews per round. A total of 20–30 interviews are recommended to ensure thematic saturation (35).

**Table 2:** Examples of verbal probes utilized during the cognitive interview.

Verbal probe type	Scripted probing	Sample questions
Comprehensive probes	What do you think the question is asking?	What do you think this sentence means? Please explain it in your own words.
		What do you think this word means in this context?
Retrieval probes	Tell me what you are thinking?	What did you recall when you answered this question?
Comfort probes	Are there any questions that make you uncomfortable?	Are there any words or items that make you uncomfortable?
Observational probes	Why did not you answer the question?	Why did not you answer the question?
		Why did you hesitate to answer this question?
General probes	What do you think of the questionnaire?	What do you think of the questionnaire (design, font, length, etc.)?

#### 2.1.7 Step 7: reliability and validity test

After linguistic validation, the Chinese version of TEMS was administered to eligible older adults within the Chinese community to further verify its reliability and validity. To allow cross-validation (36), we enrolled 249 participants, although a sample size of 200 participants would have been sufficient for confirmatory factor analysis (CFA) (37).

### 2.2 Setting and participant recruitment

The study was conducted from February to June 2023 in Changsha communities, China. Convenience sampling was used to recruit participants, who were 60 years or older and had no enteral or parenteral nutrition supplements. Informed consent was obtained from all participants, and interviews were carried out face-to-face by an experienced researcher (WL) in a quiet room in the communities of Hunan Province. Following the guidelines, participants for each round of interviews were distinct, meaning that individuals interviewed in one round did not overlap with those in subsequent rounds (38).

.The inclusion criteria were as follows: (1) participants aged 60 years or older, (2) individuals capable of effective communication and able to independently complete the questionnaire, (3) voluntary participation confirmed by signed informed consent, and (4) no enteral or parenteral nutrition supplements or voluntary restrictive diets within 1 month before the assessment. The exclusion criteria included older adults in the acute stage of any disease that may adversely affect nutritional status, energy intake, or nutrient absorption.

### 2.3 Data analysis

This study conducted data sorting and results reporting following CIRF (33). The interview process was audio recorded, transcribed in time by one researcher (WL), and reviewed by other researchers (CX and JD). Five probe categories of cognitive interviewing were considered as individual themes for data analysis and were listed separately in an Excel sheet. Then, interview transcripts were entered into NVivo qualitative software and analyzed for cognitive requirements and linguistic issues. Investigator triangulation was used for data analysis to ensure the integrity and reliability of research results, which involved two researchers independently analyzing the same interview transcripts, avoiding the unilateral observation and understanding of a single researcher, to ensure higher reliability of the research results (34, 39).

### 2.4 Ethical statement

This study was approved by the Nursing and Behavioral Medicine Research Ethics Committee of Xiangya Nursing School of Central South University in China (E202320). Permission and authorization were obtained from the original scale developer to translate the TEMS into Chinese. Informed consent was obtained from all participants involved in the study.

## 3 Results

### 3.1 Sample characteristics

All translated items demonstrated ≥90% concordance in the initial back-translation evaluation, meeting our predefined quality threshold; therefore, no re-translation was required. Thus, three iterative rounds of cognitive interviews were conducted, and a total of 23 older adults participated. Interviewees in each round did not coincide; that is, the participants interviewed in each round were different. There were 10 interviewees in the first round of cognitive interviews, and the participants were coded as 1. P1–1. P10 according to the interview sequence. In addition, eight interviewees in the second round were coded as 2. P1–2. P8, and five interviewees in the third round were coded as 3. P1–3. P5 (Table 3). The final sample included 10 women and 13 men, ranging from 60 to 78 years old (mean: 67.217 years). Most of the participants were less educated (junior high school or below, 60.87%).

**Table 3:** Sociodemographic characteristics of participants in cognitive interviews.

Category of different variables	Round 1 (*n* = 10)	Round 2 (*n* = 8)	Round 3 (*n* = 5)
Sex			
Female	1	5	4
Male	9	3	1
Age			
60–65	5	3	2
66–69	1	3	–
≥70	4	2	3
Education level			
Primary school or below	2	2	2
Junior high school	4	3	1
Senior high school	3	1	1
Junior college	1	2	1
Occupational categories			
Farmer	2	-	-
Worker	8	6	5
Enterprise	–	2	–
Marital status			
Married	9	8	3
Widowed	1	–	2
Living situation			
Live alone	–	–	2
Live with children	2	1	3
Live with spouse	5	2	-
Live with children and spouse	3	5	-

### 3.2 First-round cognitive interview

In this round, 10 participants (1. P1–1. P10) were involved and pointed out that there were ambiguities in the wording of 8 items, as well as indicated that font size and scale layout needed improvement. Based on the interview results, the research team analyzed the frequency, suggestions, and content of the questions raised by the participants in response to the questionnaire items. After discussion and modification, specific modification plans are detailed in Table 4. The modified TEMS scale was used for the second-round cognitive interview.

**Table 4:** First-round cognitive interview results (*n* = 10).

Original item	Frequency	Verbal probe type	Interview findings	Modification plan	Revised item
(Introduction) I eat certain foods because:	6	Observational probes	Researchers observed that less educated respondents needed to pause and reread the sentence in question or take breaks to think. After completing the questionnaire, the researchers inquired about the reasons behind this behavior. The respondents stated that they found the sentence’s logical structure difficult to understand in Chinese. (1. P1–P3, 1. P7, 1. P9, and 1. P10)	After the research team discussion, the sentence was modified to be more conversational and easier to understand, ensuring the meaning remained unchanged.	The reason I eat is:
1. Because I’m very hungry.	4	Comprehensive probes	The respondents’ understanding of “very hungry” is: “Because I am very hungry, I cannot bear to eat food”. (1. P4, 1. P6) “I often eat frequently, although I’m just a little bit hungry.” (1. P1, 1. P9)	There was a misunderstanding bias. After the research team discussion, the original meaning of this sentence is to eat out of hunger motivation, but it does not emphasize the degree of hunger. Thus, “very” was deleted.	Because I’m hungry.
6. Because it’s low calorie.	6	Comprehensive probes	Some respondents with low education levels said they could not understand the meaning of “calories” in Chinese. (1. P2–P3, 1. P10)	After the discussion, the word “low-calorie” in this sentence was too specialized in Chinese, so a statement was added to illustrate it.	Because it is a low-calorie food. (It is not easy to get fat.)
9. Because it’s popular.	3	Comprehensive probes	The respondents’ understanding of “popular” was “internet celebrity food,” which was recommended online. (1. P4–P6)	There was a misunderstanding related to the term “popular” in this context, which refers to food that people around them recommend or love to eat. After a discussion, the research team added a statement to further clarify the “popular” concept.	Because it is so popular. (People around are eating it.)
11. To meet the needs of certain occasions.	3	Comprehensive probes	Respondents said, “I do not understand what specific occasions refer to. Could you give an example?” (1. P2, 1. P7, and 1. P9).	After discussing with the research team, these words were added for clarification.	To meet the needs of certain occasions (e.g., social engagement, parties)
18. Because it brings a pleasant feeling of fullness.	5	Comprehensive probes	Low-educated respondents said, “Although I could understand the sentence’s meaning, I suggested modifying the word “pleasure,” which was not easy to understand in Chinese (1. P1–P3, 1. P10).” Suggest replacing “pleasant” with “makes me happy” (1. P4)	After discussion by the research team, the word “pleasure” was too formal in Chinese, so it was changed to a colloquial expression.	Because eating it makes me feel full and happy.
19. Because it would be rude not to eat.	2	Comprehensive probes	Respondents indicated that they would like to give examples of “impolite” behavior (1. P1–P2).	After the discussion, examples were provided in brackets to help the subjects understand, in line with Chinese traditional culture.	Because it would be rude not to eat (e.g., someone else serves me food).
24. Because of its attractive appearance (e.g., outer packing).	2	Retrieval probes	Respondents said, “I usually buy food in the market if it looks good, I will buy it, even if it is not packaged (1. P4, 1. P7).”	After the discussion, it is customary in Chinese culture to buy vegetables and fruits in the market, some of which do not have outer packaging. Thus, the word “presentation” was added for further explanation.	Because it is attractive (such as presentation, and packaging).
39. To “kaolao” myself.	4	Comprehensive probes	Respondents found it difficult to understand the meaning of “kaolao” in Chinese which meant “reward” in English (1. P2–P3). The word “kaolao” is too formal (1. P8).	After discussion, “kaolao” was too written in Chinese culture, and the more understandable word “jiangli” in Chinese was changed. (“kaolao” and “jiangli” in Chinese both mean “reward” in English.)	To “jiangli” myself.
Scale overall font size, and layout.	3	General probes	Subjects reported a small font size and difficulty reading. “It’s difficult for me to read in this small font size.” (2. P4, 2. P6, and 2. P7).	After a literature review and discussion by the research team, the overall font of the scale was enlarged by one time, from “Song typeface small 4” to “Song typeface times 4,” and the line spacing was changed from 1.0 times to 1.5 times.	Scale font changed to “Song typeface times 4,” 1.5 times line spacing.

### 3.3 Second-round cognitive interview

Eight participants (2. P1–2. P8) were recruited for the second-round cognitive interview after the initial revision of the TEMS scale. Notwithstanding these adjustments, participants still raised questions about identifying ambiguities within certain item phrasings. Utilizing the data derived from the second-phase cognitive interview, the research team conducted a rigorous analysis of the frequency and specifics of participants’ suggestions related to particular items. These findings were subsequently deliberated upon and integrated into the scale’s iterative refinement. A detailed exposition of the identified issues and the corresponding revisionary strategies is documented in Table 5. Following this interview, third-round cognitive interviews were conducted, employing the newly optimized TEMS scale.

**Table 5:** Second-round cognitive interview results (*n* = 8).

Original item	Frequency	Verbal probe type	Interview findings	Modification plan	Revised item
5. Because I feel frustrated.	4	Observational probes	The researchers observed that respondents with lower education levels paused slightly when answering this question. After completing the questionnaire, the researchers were asked for the reasons. Respondents said, “The word ‘frustrated’ was not common in Chinese daily life, and it was difficult to understand its meaning for low-educated people like me (2. P4-P7).”	After discussion, “frustrated” was written more in Chinese culture and was difficult to understand. The wording was modified to a colloquial expression to ensure the same semantics.	Because I feel defeated.
27. For accompanying meals.	3	Comprehensive probes	Respondents indicated that the sentence was not easy to understand and suggested modifying the word order of the sentence and the word “accompany” (1. P1–P2, 1. P10).	After discussing it, we realized that the sentence was too formal in Chinese culture, so we adjusted the word order and changed it to a more casual expression.	To have someone to eat with.
34. Because it can make the atmosphere of social gatherings more comfortable.	3	Comprehensive probes	Respondents expressed a desire to provide examples of specific behaviors that “create a more comfortable atmosphere” (1. P1–P2, 1. P4).	After the discussion, examples are given in brackets to make the contents easier for the subjects to understand.	Because it can make the atmosphere of social gatherings more comfortable (e.g., drinking at parties)
38. Because it’s low-fat food.	3	Comprehensive probes	Respondents indicated that the term “low-fat food” was not commonly used in Chinese daily communication and was somewhat difficult to understand (2. P5–P7).	After discussion, a simpler expression was revised on the premise of keeping the semantics unchanged.	Because it contains less fat.

### 3.4 Third-round cognitive interview

Five participants (3. P1–3. P5) were assembled, employing the scale refined following the second-round discussions for the third-round cognitive interview. Feedback revealed that participants generally found the scale items to be precise and intelligible, with phrasing consistent with traditional Chinese cultural values. No additional inquiries arose during the interviews, indicating a saturation of cognitive data. Consequently, the cross-cultural adjustment of the TEMS for the Chinese context was successfully achieved.

### 3.5 Reliability and validity of the TEMS Chinese version

The Chinese version of TEMS was used on 249 eligible older adults living in the community for testing reliability and validity. The results showed that Cronbach’s *α* coefficient was 0.772, the split-half coefficient was 0.871, the content validity index (S-CVI) was 0.930, and the range of the item content validity index (I-CVI) was 0.500–1.000. Additionally, the results of the exploratory factor analysis indicate that Bartlett’s sphericity test (*X^2^* = 5219.724, *df* = 990, *p* < 0.001) and the Kaiser–Meyer–Olkin (KMO) value is 0.799, exceeding 0.600. Fifteen common factors were extracted, with a cumulative contribution rate of 73.535%, including likes, habits, needs and hunger, health, convenience, pleasure, traditional diet, natural concern, social interaction, price, visual appeal, weight control, emotional regulation, social norms, and social image. The loading value of each item ranges from 0.446 to 0.901 (Table 6), exceeding 0.400, confirming that reliability and validity are good.

**Table 6:** Chinese version of TEMS items’ factor loadings.

Items	1	2	3	4	5	6	7	8	9	10	11	12	13	14	15
Q16	0.737														
Q22	0.635														
Q39	0.719														
Q4		0.811													
Q21		0.842													
Q33		0.831													
Q1			0.480												
Q18			0.794												
Q36			0.600												
Q9				0.818											
Q31				0.901											
Q42				0.861											
Q7					0.758										
Q28					0.788										
Q41					0.787										
Q6						0.828									
Q13						0.725									
Q38						0.780									
Q19							0.678								
Q25							0.718								
Q32							0.763								
Q2								0.737							
Q3								0.459							
Q23								0.547							
Q11									0.628						
Q14									0.855						
Q43									0.747						
Q8										0.489					
Q17										0.621					
Q44										0.702					
Q24											0.710				
Q30											0.724				
Q35											0.660				
Q20												0.862			
Q26												0.446			
Q29												0.834			
Q5													0.844		
Q12													0.807		
Q37													0.525		
Q10														0.663	
Q40														0.735	
Q45														0.610	
Q15															0.698
Q27															0.465
Q34															0.850

1 pleasure, 2 conveniences, 3 demand and hunger, 4 social image, 5 price, 6 weight control, 7 social norms, 8 likings, 9 traditional diets, 10 health, 11 visual appeals, 12 natural attention, 13 emotional regulation, 14 habits, 15 sociability.

## 4 Discussion

This study adhered rigorously to Brislin’s classical translation model to translate TEMS into Chinese, involving translation, back-translation, and expert consultation (32). High-quality translation and linguistic validation are the basis of psychometric and statistical testing (34). Nevertheless, practical data collection reveals challenges, such as participants with lower educational backgrounds having a limited understanding of the scale content, which may lead to misinterpretations and incorrect responses, thus deviating from the information the researchers intended to gather (40). These issues distort the data and compromise the quality of the scale. Researchers identify these as response errors, a significant barrier to the effectiveness of surveys, as designers often overestimate respondents’ comprehension of the content (41).

In this study, cognitive interviews were conducted to evaluate whether participants’ judgments of the content aligned with the original intent or introduced ambiguities. Subsequent refinements were made to ensure the content was accurate and conveyed in Chinese clearly, consistent with traditional Chinese cultural norms. Previous studies have shown that cognitive interviewing is valuable for understanding respondents’ comprehension and interpretation of questionnaires (42), a critical method for testing and refining questionnaires during cross-cultural linguistic adaptation (43). This process effectively minimized cultural discrepancies and resolved response errors (44, 45).

Most participants in the cognitive interviews had lower educational levels (junior high school level or below). Previous studies have shown that targeting respondents with lower educational levels in cognitive interviews is more effective (46). If this group accurately comprehends the scale content, it suggests that the scale is both intelligible and accurately conveys its intended meaning, thereby enhancing its content validity (46). This study identifies a preference among respondents, particularly those with lower educational levels, for clear explanations, concise presentations, straightforward logical sequences, and colloquial language. Our findings indicate that researchers often perceive terms deemed easy to understand as difficult to read by participants. Consequently, written words and technical terms in the original language were translated into more colloquial words in the Chinese version; these were not commonly used in Chinese daily communication and were somewhat difficult to understand for low-educated participants. In addition, we found that degree adverbs should be used with caution in sentence expressions, which tend to lead to response errors. Furthermore, providing examples significantly enhances participants’ comprehension of the scale items. Respondents prefer adding examples alongside technical terms to facilitate their understanding of the intended meaning. Additionally, our study reveals that a text format featuring 1.5 line spacing and a font size of 14 in SimSun font is deemed more readable and suitable for older participants. This preference likely arises from age-related visual impairments, which make simpler formatting and larger fonts more accessible for older respondents.

The TEMS factor structure demonstrated cross-cultural consistency, with our Chinese version replicating the same 15-factor solution as the Brazilian adaptation (6). Both studies achieved satisfactory psychometric properties (Cronbach’s *α* = 0.772 vs. 0.60–0.94 in Brazil), confirming measurement robustness across cultures. Notably, cultural variations emerged in motive prominence—traditional diet factors loaded strongly in our Chinese sample, while sociability motives were more salient in Brazil. These findings suggest that while TEMS captures universal eating motivations, their relative importance may reflect cultural values.

Meanwhile, our study has some limitations. As a dissertation project constrained by time limitations, this study prioritized establishing internal consistency and construct validity. Future research should incorporate test–retest assessments to fully address temporal stability according to Aldridge et al.’s comprehensive framework (47). Besides, although convenience sampling facilitated efficient participant recruitment, we acknowledge that this approach may affect the generalizability of our findings. The sample may not fully represent the broader population due to potential selection biases inherent in this method. We suggest that future studies employ probability sampling methods. In this study, cognitive interviews were conducted and analyzed in Chinese, and the subsequent translation of results and interviewee quotes into English may introduce bias. This translation process limits all researchers’ access to the original records. However, researcher triangulation was utilized to ensure the integrity of the data analysis.

## 5 Conclusion

Although time-consuming, cognitive interviewing is a crucial component of instrument development and a valuable method for identifying issues that might otherwise remain undetected and compromise the instrument’s effectiveness. It allows for the detection of linguistic and cultural nuances in cross-cultural instrument adaptation. Feedback from participants on question clarity and overall usefulness enabled researchers to make immediate adjustments. This iterative process enhanced the face validity of the instrument, which is expected to increase respondents’ willingness to participate and improve the instrument’s practical utility. Following further quantitative testing, the finalized TEMS will offer medical professionals an effective tool for assessing eating motivation among older Chinese adults.

## Data Availability

To protect participants’ privacy, the raw data that supports the conclusions of this article will be made available by the authors exclusively for legitimate research purposes, ensuring adherence to ethical standards.
