# Correction: Cross-cultural translation and linguistic validation of the eating motivation survey among older adults in the Chinese context

**DOI:** 10.3389/fnut.2025.1676552

**Published:** 2025-08-12

**Authors:** Lina Wu, Xi Chen, Hui Feng, Shouzhen Cheng

**Affiliations:** ^1^The First Affiliated Hospital of Sun Yat-sen University, Guangzhou, China; ^2^School of Nursing, Sun Yat-sen University, Guangzhou, China; ^3^Xiangya School of Nursing, Central South University, Changsha, China

**Keywords:** eating motivation, food choice, linguistic validation, cognitive interview, healthy aging

In the published article, the author, Shouzhen Cheng, was erroneously assigned to affiliation 2 School of Nursing, Sun Yat-sen University, Guangzhou, China. The correct affiliation is 1 The First Affiliated Hospital of Sun Yat-sen University, Guangzhou, China.

The original article has been updated.

